# Leaf Coloration in *Acer palmatum* Is Associated with a Positive Regulator *ApMYB1* with Potential for Breeding Color-Leafed Plants

**DOI:** 10.3390/plants11060759

**Published:** 2022-03-12

**Authors:** Sujing Sun, Qiang Zhang, Yongfan Yu, Jianyuan Feng, Changlai Liu, Jiading Yang

**Affiliations:** 1 Co-Innovation Center for Sustainable Forestry in Southern China, College of Biology and the Environment, Nanjing Forestry University, Nanjing 210037, China; sunsujing2020@163.com (S.S.); zhangqiang@njfu.edu.cn (Q.Z.); yuyfworking@163.com (Y.Y.); fengjianyuan2002@163.com (J.F.); lcl2012@njfu.edu.cn (C.L.); 2 School of Health, Jiangsu Food and Pharmaceutical Science College, Huai’an 223005, China; 3 Bamboo Research Institute, Nanjing Forestry University, Nanjing 210037, China

**Keywords:** *Acer palmatum*, *ApMYB1*, anthocyanin accumulation, autumnal senescence, ectopic transformation, tobacco

## Abstract

Anthocyanin biosynthesis and accumulation is closely associated with tissue/organ coloring in plants. To gain insight into the physiological and molecular mechanisms of leaf coloring in *Acer palmatum*, a deciduous tree during autumnal senescence, we first investigated concentration dynamics of pigments (i.e., chlorophyll, carotenoid and anthocyanin) in leaves with differential coloring. It was found that compared to green leaves (GN), anthocyanins were accumulated actively in semi-red (SR) and total-red (TR) leaves, accompanied with chlorophyll and carotenoid degradation. Then transcriptional profiling on GN and SR leaves identified thousands of transcripts with differential expression in SR compared to GN leaves. An annotation search showed that the entire flavonoid/anthocyanin biosynthesis pathway from the production of naringenin chalcone to modification of flavonoid backbone was extensively activated at the transcriptional level in SR leaves. Phylogenetic analysis of putative MYB proteins identified ApMYB1 as a putative regulator promoting anthocyanin biosynthesis. Expression of *ApMYB1* in leaves was induced by exogenous hormones including abscisic acid. Stable overexpression of *ApMYB1* in tobacco resulted in leaves with higher accumulation of anthocyanins. Collectively, our results identified *ApMYB1* as a positive regulator associated with leaf coloring in *Acer palmatum* during autumnal senescence, which may be regarded a potential target for breeding color-leafed plants.

## 1. Introduction

For deciduous trees with autumnal leaf coloring, two physiological components are essential: chlorophyll degradation and/or anthocyanin biosynthesis, both of which mainly happen during leaf senescence [1]. Degreening is the most obvious event of leaf senescing and chlorophyll content is often used as a marker to determine the extent of senescence processing. During procedure of leaf senescence, with chlorophyll being catalyzed into colorless metabolites, other pigments such as carotenoids and anthocyanins become visible, thus rendering leaves colorful in autumn [2,3].

Unlike carotenoids which are present in leaves all year round, anthocyanins are mainly de novo synthesized during leaf autumnal senescence [4]. The pathway of anthocyanin biosynthesis has been studied in many plant species and most of the genes responsible for enzymatic steps have been identified. Anthocyanins and other flavonoids are synthesized initially through the phenylpropanoid pathway, through which phenylalanine is converted into 4-coumaroyl-CoA by phenylalanine ammonia-lyase (PAL), cinnamic acid 4-hydroxylase (C4H) and 4-coumarate:coenzyme A ligase (4CL) sequentially [5]. Flavonoid pathway starts with the condensation of one molecule of 4-coumaroyl-CoA and three molecules of malonyl-CoA catalyzed by chalcone synthase (CHS) to form naringenin chalcone, which then be catalyzed by chalcone isomerase (CHI), flavanone 3-hydroxylase (F3H) (or flavonoid 3′-hydroxylase, F3′H) to produce dihydroflavonols [6]. Then dihydroflavonols are converted by dihydroflavonol 4-reductase (DFR), leucoanthocyanidin oxygenase (LDOX/ANS) to anthocyanidins which are then glycosylated by uridine diphosphate (UDP)-glucose: flavonoid-O-glycosyl-transferase (UFGT) to produce anthocyanins. Anthocyanins can be further catalyzed by O-methyltransferases (OMTs) or acyltransferases (ATs) to produce modified anthocyanins [3,7]. Different forms of anthocyanins may have differential color, solubility, reactivity and thus physiological activity [5]. For example, the antioxidant activity of anthocyanins is increased markedly by diacylation, but was reduced by 5-glycosylation [8].

In addition to above mentioned structural biosynthetic genes, transcription factors play important roles in modulating anthocyanin biosynthetic pathway activity. The well-known MBW regulatory complex, which consists of DNA-binding R2R3 MYB transcription factors and MYC-like basic helix-loop-helix (bHLH) and WD40-repeat proteins, has been identified to regulate structural genes of anthocyanin biosynthesis in multiple species [9,10,11]. Among three members of MBW complex, MYB and bHLH have relatively more specificity than WD40 proteins to determine the subset of activated genes [12]. In Arabidopsis, R2R3-type MYB transcription factors [i.e., PAP1/MYB75, PAP2/MYB90, MYB113, and MYB114] positively regulate anthocyanin biosynthesis in vegetative tissues [13,14,15]. A fairly large number of MYB members positively regulating anthocyanin production have been identified in many other plant species (see review [16]). Additionally, some R2R3-type and R3-type MYB proteins have been identified as repressors of anthocyanin biosynthesis by either repressing the activity of the MBW complex or the expression of components of MBW complex [17]. In Arabidopsis, a small R3-MYB protein MYBL2 can bind directly to bHLH member of MBW complex to suppress expression of *DFR*, thus inhibiting anthocyanin accumulation [18,19]. Ectopic expression of a R2R3 MYB transcription factor *NtMYB2* from Chinese narcissus (*Narcissus tazetta* L.) in tobacco significantly reduced the floral pigmentation by repressing transcript levels of *UFGT* [20]. Both positive and negative MYB proteins together may allow fine-tuning of anthocyanin biosynthesis in various tissue types and in response to developmental, environmental, and hormonal cues. It is thus believed that MYB TFs may play a dominant role in the regulatory network of anthocyanin biosynthesis [16].

Multiple phytohormones have been indicated to regulate anthocyanin biosynthesis. Ethylene production is essential for the ripening of climacteric fruits with inducing respiratory burst at the onset of ripening and coloring. Exogenous ethylene treatment markedly induced apple fruit coloration [21]. In non-climacteric strawberry fruits, either a decrease in abscisic acid (ABA) level or silencing of a putative ABA receptor resulted in inhibition of ripening and anthocyanin accumulation [22]. Cytokinin could enhance sugar-induced anthocyanin biosynthesis in Arabidopsis [23]. Additionally, gibberellic acid and jasmonic acid signaling pathways work antagonistically in regulating anthocyanin biosysnthesis through liberating/sequestering MYBL2/JAZs, suppressors of MBW complex, by DELLA proteins in Arabidopsis [24]. However, the more details about hormonal regulation of anthocyanin biosynthesis remain largely unknown.

Trees of *Acer* genus (belonging to *Sapindaceae* family) are distributed widely in the northern hemisphere, primarily East Asia, North America, and Europe [25]. Many *Acer* species, including *Acer palmatum,* are grown as ornamentals due to spectacular leaf coloration especially in autumn [25]. However, few studies were conducted to elucidate the physiological or molecular mechanisms of leaf coloration in *Acer palmatum* during autumnal senescence. We observed that the leaves of different layers in the canopy of *Acer palmatum* trees always showed a differential coloring process. Considering that environmental factors except light intensity/quality were almost same for an individual tree, it is postulated that altered light signals due to canopy shading may be responsible for leaf differential coloration in *Acer palmatum*. However, the associated physiological and molecular mechanism is unknown yet. In the present study, we investigated pigment levels in *Acer palmatum* leaves with differential coloration (green, semi-red, and total-red) on the same branch at leaf-coloring stage. We further identified differentially expressed transcripts (DETs) between green and semi-red leaves which may be associated with anthocyanin biosynthesis in semi-red leaves. A MYB gene (*ApMYB1*) was then functionally characterized for its function in anthocyanin production.

## 2. Results

### 2.1. Concentrations of Chlorophyll, Carotenoid and Anthocyanin in Various Leaves

**Figure 1:**
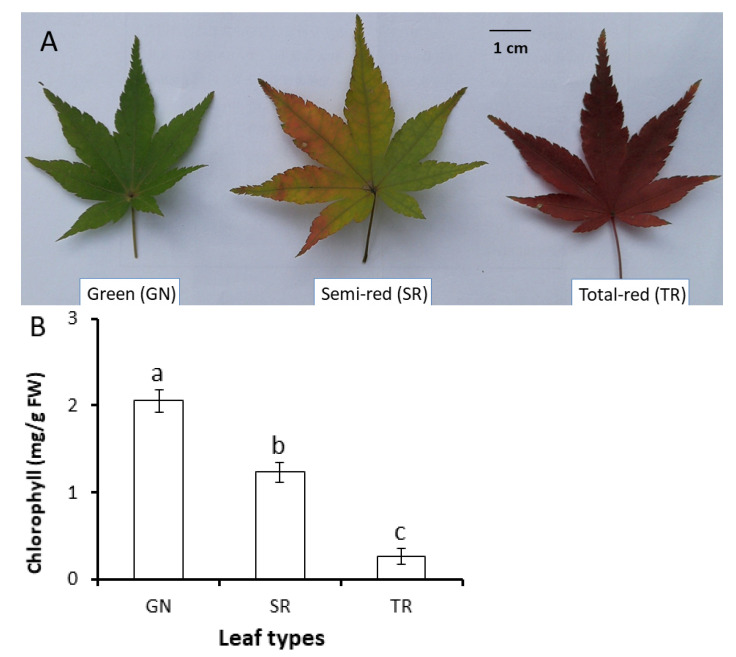

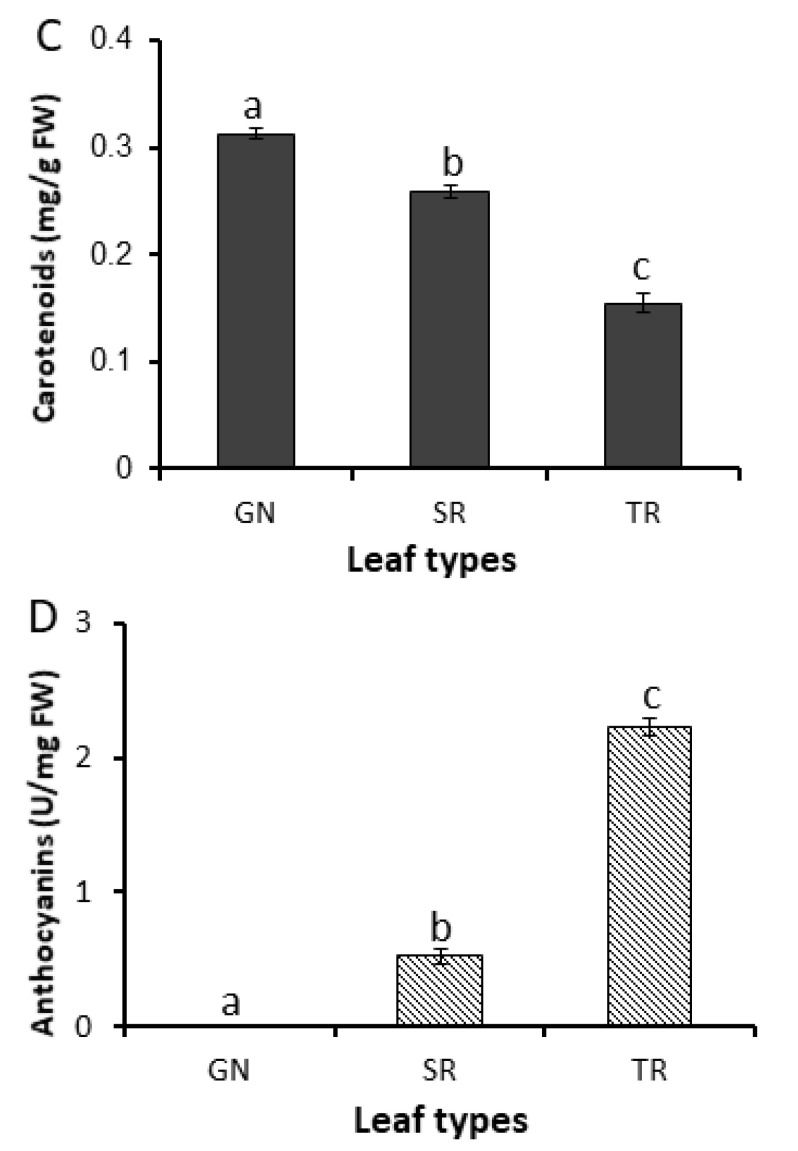
Phenotype and pigment concentrations of three types of leaves (GN, green; SR, semi-red; TR, total-red). (**A**) Phenotype of representative leaves with differential color on the same branch when samples were collected. (**B**) concentration of total chlorophyll, (**C**) carotenoids and (**D**) anthocyanins in leaves. One unit of anthocyanin concentration was expressed as a change of 0.1 absorption of methanolic extracts (U/mg FW) (see Section 4). Values are means ± SD (*n* = 3). The statistical significance among means ± SD (*n* = 3) among three types of leaves was indicated by different lower letters at *p* < 0.05.

Consistent with the differential visual phenotype (Figure 1A), GN leaves contained the highest chlorophyll concentration as 2.06 mg/g FW, while chlorophyll concentration was 1.23 mg/g FW in SR leaves and 0.26 mg/g FW in TR leaves, as 60% and 13% of chlorophyll concentration in GN, respectively, indicating an active chlorophyll degradation in SR and TR leaves (Figure 1B). Carotenoid concentration was highest in GN leaves as 0.31 mg/g FW, and it decreased to 83% in SR and 49% in TR compared to that in GN, respectively (Figure 1C). While the anthocyanin in GN was 0.017 Unit, it was increased about 28 folds in SR (as 0.525 Unit) and increased 131 folds in TR (as 2.23 Unit) (Figure 1D). Thus, it meant that anthocyanin biosynthesis, accompanying with chlorophyll and carotenoid degradation, was actively processed in SR and TR leaves and turning red of *Acer palmatum* leaves in autumn may be mainly due to anthocyanin accumulation.

### 2.2. Transcriptome Analysis and Differentially Expressed Transcripts Putatively Responsible for Flavonoid/Anthocyanin Biosynthesis

**Figure 2:**
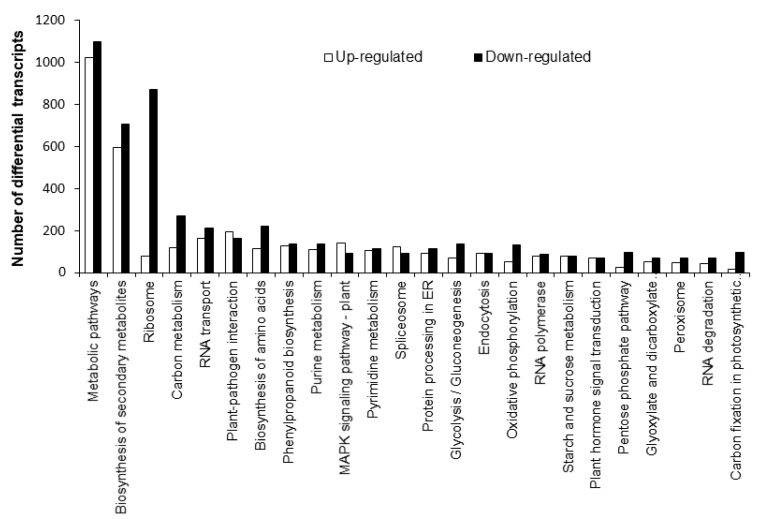
Various pathways and number of differentially expressed transcripts (DETs) identified by KEGG (Kyoto Encyclopedia of Genes and Genomes) Enrichment analysis. DETs in each pathway were separated as up- and down-regulated to estimate the overall activity of pathway as activated or repressed.

RNA-Seq on 6 cDNA libraries of GN and SR leaves (each with three biological replicates) generated totally 397.09 million clean reads. A total of 88,893 putative transcripts were assembled and 11,829 transcripts were classified as differentially expressed transcripts (DETs) based on multiple criteria. Among DETs, 6407 transcripts were up-regulated (expression fold change ≥ 2.0) and 5422 transcripts were down regulated (fold change ≤ 0.5) in SR leaves compared to GN leaves (Supplementary Table S1). To inspect the primary functions of DETs, KEGG (Kyoto Encyclopedia of Genes and Genomes) Enrichment analysis was performed (Supplementary Table S2). Among 134 pathways, metabolic pathways and biosynthesis of secondary metabolites were the top two enriched pathways with largest numbers of DETs (Figure 2), indicating a substantial variation of metabolic activity in SR leaves compared to GN leaves. Notably, the third top pathway was Ribosome (Translation), in which 874 out of 950 DETs were down-regulated, possibly indicating an attenuated protein biosynthesis in SR leaves. Interestingly, consistent with known physiological changes during leaf senescence [26], pathways such as carbon fixation in photosynthetic organisms (16 up/95 down), photosynthesis (6 up/36 down) and pentose phosphate pathway (26 up/98 down) were found to contain much more down-regulated genes, while ascorbate and aldarate metabolism (44 up/13 down), phenylalanine metabolism (40 up/13 down), proteasome (32 up/10 down) and flavonoid biosynthesis (29 up/9 down) had more up-regulated transcripts. In addition, transcripts for fatty acid degradation (25 up/8 down), sphingolipid metabolism (24 up/6 down) and ether lipid metabolism (26 up/4 down) indicated that these pathway may be activated in SR leaves (Supplementary Table S2).

Furthermore, the up-regulated transcripts which may be putatively involved in flavonoid/anthocyanin biosynthesis in SR leaves were identified based on functional annotations (Supplementary Table S3), including those putatively encoding 7 CHS (chalcone synthase), 2 CHI (chalcone isomerase), 7 F3H (flavanone 3-hydroxylase), 5 DFR (dihydroflavonol 4-reductase), 3 FLS (flavonol synthase), 2 ANS/LDOX (anthocyanidin synthase/leucocyanidin oxygenase), 1 LAR (leucocyanidin reductase), and 1 F3′H (flavonoid 3′-hydroxylase). Additionally, 30 transcripts annotated as encoding glucosyltransferase and 17 transcripts encoding O-methyltransferase were up-regulated in SR, among which certain members actually participating in modifying anthocyanin formation remain to be identified experimentally. These results indicated that the entire flavonoid/anthocyanin biosynthesis pathway from production of naringenin chalcone to modification of flavonoid backbone was extensively activated at the transcriptional level in SR leaves. The expression levels of 20 selected transcripts in GN and SR putatively involved in flavonoid/anthocyanin biosynthesis were then measured by qPCR and results confirmed their up-regulation in SR leaves (Supplementary Figure S1), being consistent with RNA-seq data (Supplementary Table S3).

### 2.3. Transcripts Encoding Transcription Factors and Identification of a Putative Target MYB Transcript (ApMYB1)

**Figure 3:**
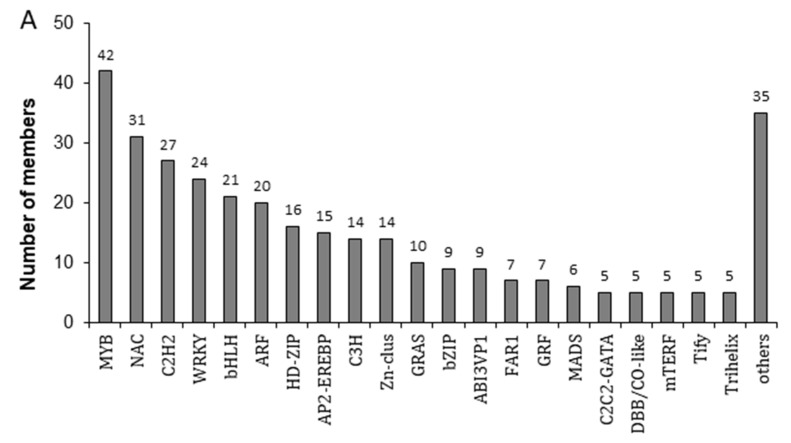

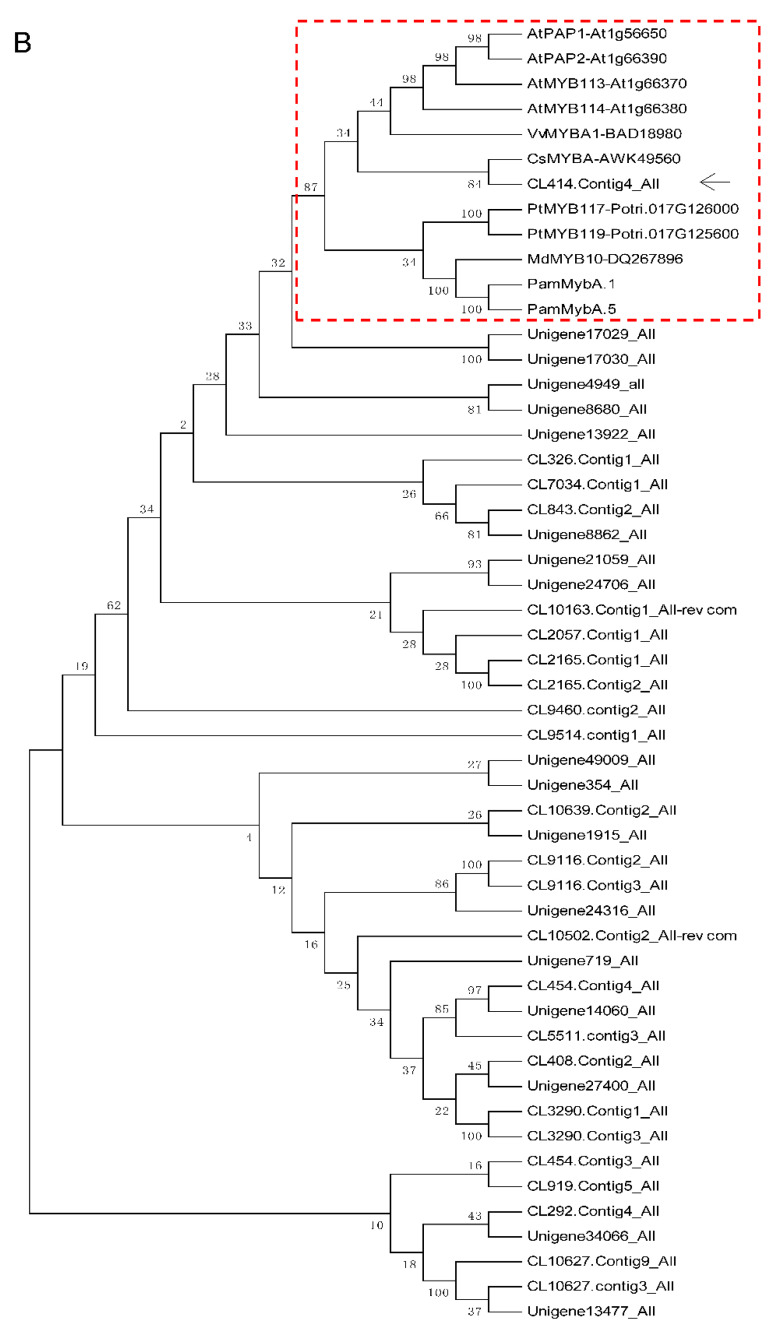
Putative TF families encoded by differentially expressed transcripts and phylogenetic analysis of MYB sequences. (**A**) Annotated TF families of differentially expressed transcripts putatively encoding transcription factors. (**B**) Phylogenetic analysis of deducted peptide sequences of *Acer palmatum* 42 differentially expressed MYB transcripts and 11 reference MYBs with known function of promoting anthocyanin biosynthesis. The sub-branch of phylogenetic tree containing 11 reference MYBs and one *Acer palmatum* MYB (CL414.Contig4_All, as indicated by a black arrow, named ApMYB1 thereafter) is enclosed in the dashed rectangle. The intact phylogenetic tree was obtained by aligning peptide sequences of all 42 differentially expressed MYB transcripts with eleven reference MYBs with known function of promoting flavonoid/anthocyanin biosynthesis in *Arabidopsis thaliana* (At), *Vitis vinifera* (Vv), *Malus domestica* (Md), *Citrus sinensis* (Cs), *Prunus americana* (Pam) and *Populus trichocarpa* (Pt).

Considering that transcription factors play the pivotal role in coordinating expression of flavonoid pathway genes [6], a total of 332 putative transcripts encoding transcription factors among 11,829 DETs were identified (Supplementary Table S4) and were classified into tens of families (Figure 3A), among which the top six with larger members were MYB (42 transcripts), NAC (31), C2H2 (27), WRKY (24), bHLH (21) and ARF (20), indicating that coloring of *Acer palmatum* SR leaves may involve a complicated regulatory network, although the detailed mechanism remains to be elucidated thoroughly.

In order to decipher the regulatory mechanism of leaf coloring of *Acer palmatum*, MYB genes were considered as a starting point and investigated to identify specific members involved in regulating flavonoid/anthocyanin biosynthesis. The deducted peptide sequences of 42 differentially expressed MYB transcripts were subject to phylogenetic alignment with eleven reference MYBs with known function of promoting flavonoid/anthocyanin biosynthesis in *Arabidopsis thaliana*, *Vitis vinifera*, *Malus domestica*, *Citrus sinensis*, *Prunus* *americana* and *Populus trichocarpa*. As shown in Figure 3B, all eleven reference MYBs and one *Acer palmatum* MYB (CL414.Contig4_All, named ApMYB1 thereafter) were group in the same sub-clade of phylogenetic tree (Supplementary Table S5) and exhibited a relatively closer evolutionary relationship with MYB from *Citrus sinensis* (CsMYBA) with 53% amino acid identity.

### 2.4. Cloning of ApMYB1 and Its Expression Responding to Exogenous Treatments

**Figure 4:**
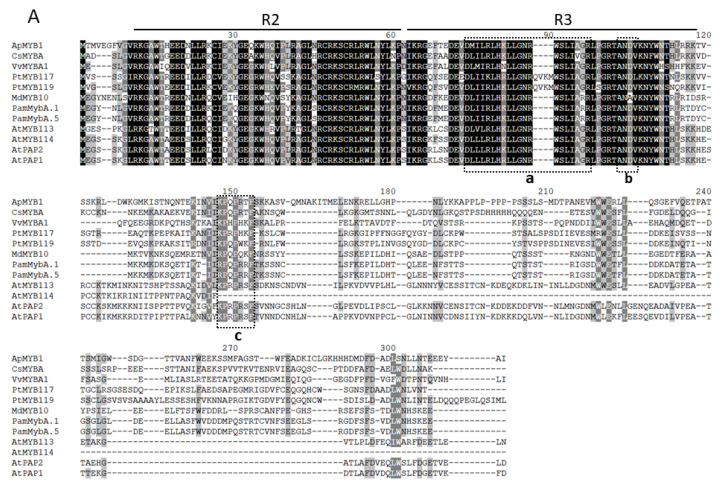

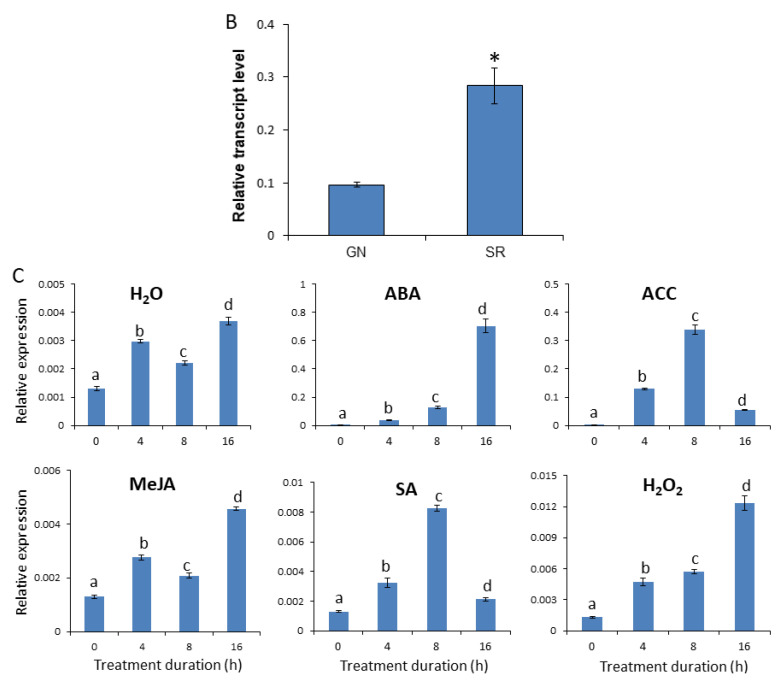
Identification of the target ApMYB1. (**A**) Alignment of ApMYB1 and eleven reference MYB TFs with known function of promoting anthocyanin biosynthesis. Black shading indicates identical amino acids. The R2 and R3 domains were indicated with black lines. The motif for bHLH binding and ANDV motif in R3 domain were enclosed with dotted rectangles a and b, respectively. The KP[Q/R]PR[S/T]F motif downstream of R3 domain was enclosed with dotted rectangles c. (**B**) The relative transcript level of *ApMYB1* in green and semi-red leaves measured by qPCR. (**C**) Responses of *ApMYB1* transcription to exogenous chemical treatments. Excised *Acer palmatum* leaves were incubated under light with H_2_O (negative control), ABA (20 μM), ACC (precursor of ethylene, 50 μM), MeJA (50 μM), SA (100 μM) and H_2_O_2_ (1%). Values in (**B**,**C**) are means ± SD (*n* = 3). In (**B**), the asterisk indicates a significant difference between transcript levels of semi-red (SR) and green leaves (GN) (*p* < 0.05). In (**C**), different lower-case letter indicates significant difference among values at different treatment durations (*p* < 0.05).

The open reading frame (ORF) of *ApMYB1* was cloned by RT-PCR and included 837 bp encoding a peptide with 278 amino acids (Supplementary Table S5). Sequence comparison showed that ApMYB1 protein contained highly conserved R2 and R3 domains in the N-terminal region, similar to those reference MYBs (Figure 4A). In R3 domain, there was the bHLH binding motif which may allow formation of a functional activation complex driving flavonoid/anthocyanin biosynthesis and the ANDV motif which was identified in anthocyanin-promoting MYBs in dicot plants [27]. In addition, the KP[Q/R]PR[S/T]F motif was also present downstream of the R3 domain [28]. Similar to RNA-Seq data, the transcript level of *ApMYB1* was about three fold higher in SR as that in GN leaves (Figure 4B), consistent with the higher anthocyanin accumulation in SR leaves (Figure 1D). These results implied that ApMYB1 may act as a positive regulator of flavonoid/anthocyanin biosynthesis in *Acer palmatum*. In addition, the effects of several senescence-promoting chemicals [i.e., abscisic acid (ABA), 1-amino-cyclopropane-1-carboxylic acid (ACC, the direct precursor of ethylene), methyl jasmonate (MJ) and salicylic acid (SA) and H_2_O_2_] on *ApMYB1* expression were tested with detached leaves of *Acer palmatum*. As shown in Figure 4C, *ApMYB1* transcription in MeJA and H_2_O showed similar pattern and magnitude, all other four chemicals could induce *ApMYB1* expression but with differential patterns. ABA and H_2_O_2_ induced continuous increasing of *ApMYB1* transcription with peaks at 16 h, while *ApMYB1* transcription peaks were at 8 h in ACC and SA treatments. Based on the expression levels of various treatments, ABA and ACC were found to have the strongest induction on *ApMYB1* transcription.

### 2.5. Ectopic Expression of ApMYB1 in Tobacco Plants Induced Anthocyanin Biosynthesis

**Figure 5:**
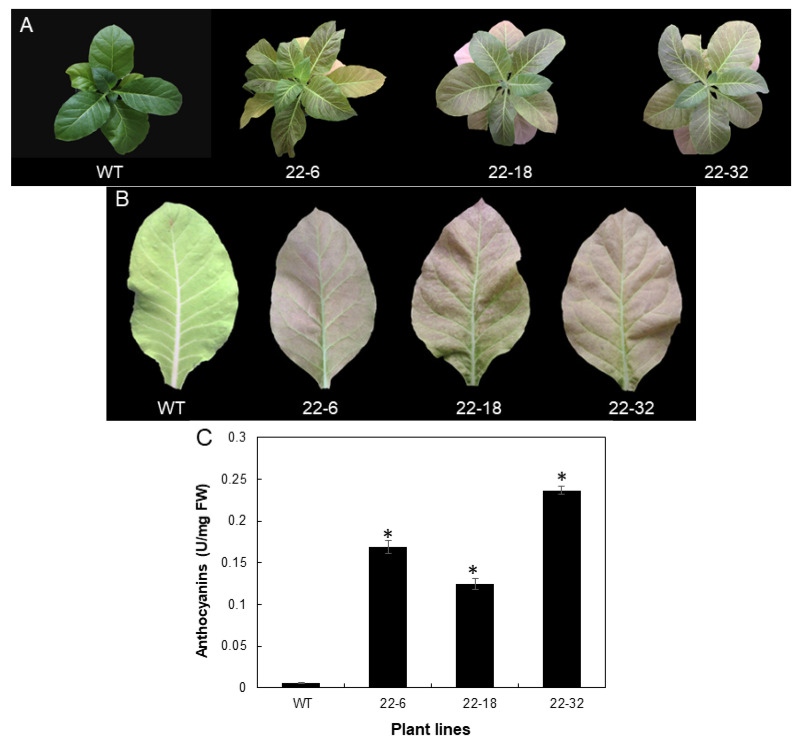
Promoted anthocyanin biosynthesis in transgenic tobacco T2 plants. (**A**) phenotype of WT and three T2 plants of transgenic line #22. (**B**) phenotype of representative leaves detached from WT and three T2 plants of transgenic line #22. (**C**) relative content of anthocyanins in leaves of WT and three T2 plants of transgenic line #22. Values in C are means ± SD (*n* = 3). Asterisks indicate significant difference between various transgenic leaves and WT at *p* < 0.05, using the Student’s *t*-test.

To confirm the deduced function of *ApMYB1* in regulating anthocyanin biosynthesis, a constitutive construct (*35S::ApMYB1*) was transformed into tobacco (*Nicotiana tabacum*). Twelve regenerated T1 plants were obtained on selection medium and 10 plants were identified as transgenic positives (Supplementary Figure S2A). Two independent T1 transgenic tobacco lines (#20 and #22) were selected for further study, based on their higher relative expression of *ApMYB1* in T1 plants (Supplementary Figure S2B). Although no obvious phenotypic difference was observed between selected transgenic T1 plants and WT, T2 seedlings of two transgenic lines showed dark color in green leaves and purple in senescent leaves (Figure 5A,B, Supplementary Figure S3A,B). Physiological measurement showed that leaves of T2 transgenic tobacco plants contained a significantly higher concentration of anthocyanins than WT leaves (Figure 5C, Supplementary Figure S3C), suggesting that overexpression of *ApMYB1* promoted anthocyanin biosynthesis in tobacco leaves.

## 3. Discussion

Changing leaf color is a common event observed in temperate deciduous trees in autumn. Three types of pigments in leaves, i.e., chlorophylls, carotenoids and anthocyanins, determine leaf color [2]. During trees’ growing season, chlorophyll is usually predominant, thus rendering greenness to leaves. However, with trees entering yearly senescence in autumn, chlorophyll is gradually degraded and other two pigments (carotenoids and anthocyanins) are then unmasked to show bright colors. While carotenoids are present in leaves throughout the growing season and their degradation is usually incomplete during leaf senescence in most plant species [29], anthocyanins are newly synthesized in senescing leaves [2].

In this study, three types of leaves with differential leaf color [i.e., green (GN), semi-red (SR) and total-red (TR)] were collected from an *Acer palmatum* tree at transition stage of leaf coloring in autumn. Pigment measurement revealed that both chlorophylls and carotenoids showed a pattern of decreasing during leaf coloration from GN to SR to TR with chlorophylls exhibiting a relatively larger rate of declining, while anthocyanins were actively biosynthesized in SR and TR (Figure 1B–D). Considering that no high-quality RNA could be extracted from TR leaves, the highest anthocyanin accumulation and lowest chlorophyll retention may together indicate the furthest extent of leaf senescence progressing in TR leaves.

Through transcriptomic analysis, 11,829 differentially expressed transcripts (DETs) were identified in SR leaves (Supplementary Table S1). KEGG (Kyoto Encyclopedia of Genes and Genomes) Enrichment analysis showed that metabolic pathways and biosynthesis of secondary metabolites were the top two enriched pathways with comparable numbers of up- and down-regulated transcripts in each pathway (Figure 2). However, for Ribosome (Translation) as the third top pathway, the number of down-regulated transcripts was more than 10-fold more than that of the up-regulated transcripts. Similarly, pathways including carbon fixation in photosynthetic organisms, photosynthesis and pentose phosphate pathways contained much more down-regulated genes. Conversely, pathways including proteasome, fatty acid degradation, sphingolipid metabolism and ether lipid metabolism contained much more up-regulated transcripts (Supplementary Table S2). The differential transcriptional activities of these various pathways were consistent with those known physiological changes during leaf senescence, i.e., fading of photosynthetic capacity, macromolecule degradation and nutrient remobilization [26]. Furthermore, consistent with anthocyanin accumulation in SR leaves (Figure 1D), up-regulated transcripts putatively encoding enzymes for anthocyanin biosynthesis were identified based on annotations and the up-regulation of selected transcripts in SR leaves was confirmed by qPCR measurement (Supplementary Figure S1). These enzymes included CHS (chalcone synthase), CHI (chalcone isomerase), F3H (flavanone 3-hydroxylase), F3′H (flavanoid 3′-hydroxylase), DFR (dihydroflavonol 4-reductase), FLS (flavonol synthase), ANS/LDOX (anthocyanidin synthase/leucocyanidin oxygenase), LAR (leucocyanidin reductase), glucosyltransferase and O-methyltransferase, indicating that every step from the formation of naringenin chalcone by CHS to the modification of flavonoid backbone by various transferases may be extensively activated at the transcriptional level in SR leaves [5]. It is of significance to experimentally characterize the exact transcripts encoding real enzymes for anthocyanin biosynthesis in *Acer palmatum* leaves in the future.

Regulatory mechanisms of anthocyanin biosynthesis at the transcriptional level have been well studied in many plants [30]. A ternary complex consisting of R2R3 MYB transcription factor, basic helix-loop-helix (bHLH) and WD40-repeat protein (known as MBW complex) has been identified to be widespread in the Eudicots for activating expression of anthocyanin biosynthetic genes [31], while other repressors may bind to MBW complex to modulate it activity [24]. In this study, 332 differentially expressed transcripts were annotated as encoding putative TFs including MYB, NAC, C2H2, WRKY and bHLH etc. in SR leaves (Figure 3A, Supplementary Table S4), possibly indicating a complicated transcriptional regulation involved in leaf coloring.

Considering that the specificity of MBW complex or the determination of downstream genes for activation is provided mainly by MYB component [32], the MYB members were selected in this study as the primary starting point to elucidate the regulatory mechanism of flavonoid/anthocyanin biosynthesis, and ApMYB1 was identified as the desired target. First, ApMYB1 was found to be in same clade of the phylogenetic tree, with all eleven reference MYBs with the known function of promoting flavonoid/anthocyanin biosynthesis (Figure 3B). Second, the ApMYB1 protein was found to have conserved R2 and R3 domain in the N-terminal (Figure 4A). Besides the bHLH binding motif allowing putative formation of a functional complex with bHLH factor, the ApMYB1 protein contains both ANDV motif and KP[Q/R]PR[S/T]F motif, which were identified in anthocyanin-promoting MYBs previously [27,28]. Third, the expression of *ApMYB1* was found to be up-regulated in SR leaves (Figure 4B). Exogenous treatments revealed that ABA and ACC (the precursor of ethylene) were the two strongest inducers of *ApMYB1* expression in leaves of *Acer palmatum* (Figure 4C). This is consistent with previous reports that ABA mediates development-dependent anthocyanin biosynthesis and coloration in *Lycium* fruits [33], and ethylene promotes anthocyanin accumulation in fruits of apple, grape berries, and plum [34]. However, how ABA or ethylene is involved in leaf coloring of deciduous trees remains elusive. These data indicated that ApMYB1, as a typical R2R3-type MYB transcription factor, is responsive to both developmental and hormonal signals and may act as a positive regulator of anthocyanin biosynthesis during leaf coloring in *Acer palmatum*.

The deduced role of *ApMYB1* was confirmed by stable overexpression in tobacco based on that T2 transgenic plants of selected two transgenic lines with higher *ApMYB1* expression produced purple color in senescent leaves (Figure 5A, Supplementary Figure S3A). Anthocyanin measurement also confirmed that anthocyanin was accumulated in transgenic leaves with tens of folds of increasing, compared to WT (Figure 5C), thus indicating that *ApMYB1* may be used for breeding color-leafed plants by genetic transformation. However, compared to transgenic poplar plants overexpressing *PtrMYB119* [35], which was previously identified as a transcriptional activator of anthocyanin accumulation, the leaf color phenotype of tobacco leaves in our study was relatively weak (Figure 5B). Considering the difference between nonwoody and woody species (tobacco versus poplar), it will be interesting to see the phenotype of a woody plant (e.g., poplar) overexpressing *ApMYB1* in future studies.

In summary, biosynthesis and accumulation of anthocyanins was accompanied by degradation of chlorophyll and carotenoids during coloring of *Acer palmatum* leaves. A complicated network involving hundreds of transcription factors may underpin the molecular regulation of leaf coloring. ApMYB1 was identified with the function of promoting anthocyanin biosynthesis. Our results advance the understanding of the physiological and molecular mechanisms of leaf coloring in deciduous trees and indicate that *ApMYB1* may be used as a potential target for breeding color-leafed plants. This study provided a valuable basis for further elucidating mechanisms of coloration of *Acer palmatum* leaves during autumnal senescence.

## 4. Materials and Methods

### 4.1. Plant Materials

A 5-year-old tree of *Acer palmatum* was selected in the campus of Nanjing Forestry University (32.1° N latitude and 118.8° E longitude), Jiangsu Province, China for material collection. The tree was exposed to all natural conditions including day-length, temperature and precipitation. The leaves in various layers of canopy which showed differential coloration were collected from an individual branch in the late November, 2017 and separated into three sets: green (GN), semi-red (SR) and total-red (TR) respectively. Each set (about 30–40 leaves) was separated randomly into three biological replicates with about 10–12 individual leaves per replicate. The leaves of each replicate were put into a 50 mL polypropylene centrifuge tube and kept in liquid nitrogen quickly. All leaf samples were then stored at −80 °C until pulverized in liquid N_2_ with mortar and pestle.

### 4.2. Spectrophotometric Measurement of Chlorophyll, Carotenoid and Anthocyanin Concentrations

Chlorophylls and carotenoids were extracted from powdered samples with 80% acetone in water and the absorptions at 663, 646 and 470 nm of extracts were determined using a Shimadzu UV-1800 UV-Vis Spectrophotometer (Kyoto, Japan). Concentrations of total chlorophyll and total carotenoids of acetone extracts were calculated respectively according to the equations as follows [36,37]:

[Chl a] (μg/mL) = 12.25 × A_663_−2.55 × A_646_

[Chl b] (μg/mL) = 20.31 × A_646_−4.91 × A_663_

[Total carotenoids] (μg/mL) = (1000 × A_470_−2.05 × [Chl a]−114.8 × [Chl b])/245

The concentrations of total chlorophyll (as Chl a + Chl b) and total carotenoids in leaves (mg/g FW) were then calculated based on sample weights and extraction volumes.

Anthocyanins were extracted with 1% methanolic HCL solution (i.e., methanol containing 1% HCl) with mild shaking overnight in the dark at 4 °C [38]. The relative anthocyanin concentration was calculated based on the absorption of methanolic extracts at 530 nm and 653 nm as (A_530_−0.24 × A_653_) [39]. One unit of anthocyanin concentration was expressed as a change of 0.1 absorption (U/mg FW).

### 4.3. Sequencing and Data Assembly

Because no high-quality RNA could be extracted from TR leaves, only GN and SR leaves (each with three biological replicates) were selected for RNA-Seq. Total RNA was isolated from leaf powder samples using a modified CTAB method [40]. The RNA samples were treated with DNase I (Promega, Madison, WI, USA), then quantity and quality of RNAs were determined by Nanodrop 2000 spectrophotometer (NanoDrop Technologies, Wilmington, ED, USA) and Agilent Bioanalyzer 2100 system (Agilent Technologies, Palo Alto, CA, USA) respectively. The mRNAs were first purified from RNA samples using poly-T oligo-attached magnetic beads and then fragmented for cDNA synthesis using random hexamer. The double-stranded cDNAs were purified and ligated to adaptors for paired-end sequencing on the BGISEQ-500 sequencing platform by Beijing Genomics Institute (BGI, Shenzhen, China).

Raw reads were filtered to remove those containing low-quality, adaptor-polluted and high content of unknown base (N), thus obtaining clean reads for downstream analyses. As there is no reference genome for *Acer palmatum*, the de novo assembly with clean reads was performed to get transcripts using Trinity [41], then TGICL was used to cluster transcripts of all sequenced samples to remove redundance [42]. The final Unigenes includes two parts: (1) the differential transcripts having >70% similarity which were clustered together were marked with same CL number and different Contig number; (2) The singletons which could not be clustered were directly marked as Unigenes.

### 4.4. Functional Annotation of Unigenes and Identification of Transcription Factors

Six functional databases, i.e., non-redundant protein database (NR), nucleotide sequence database (NT), Clusters of euKaryotic Orthologous Groups (KOG), Kyoto Encyclopedia of Genes and Genomes (KEGG), SwissProt and InterPro, were searched to assign putative functional annotations to Unigenes based on sequence similarity. To find the putative coding sequence (CDS) of each Unigene, the TransDecoder software (https://transdecoder.github.io (accessed on 11 March 2022)) was utilized and the longest open reading frame (ORF) was selected to blast with SwissProt in order to search for the Pfam protein homology sequences, thus predicting 5′ UTR, 3′ UTR and CDS. To identify Unigenes putatively encoding TFs, the ORFs were further blast against the protein domains of various plant transcription factors (TFs) as stored in PlntfDB (http://plntfdb.bio.uni-potsdam.de/v3.0/ (accessed on 11 March 2022)).

### 4.5. Identification of Differentially Expressed Transcripts (DETs) and Validation of Expression by Quantitative Real-Time PCR (qPCR)

The Bowtie2 software was used to map all the clean reads of each sample to the Unigenes [43] and RSEM program was used to calculate the expression levels [44], thus getting the FPKM (fragments per kilobase of transcript per million mapped reads) values of each transcript in three biological replicates. Those transcripts with average FPKM values greater than 1.0 in at least one set of leaf samples were considered as expressed. The differentially expressed transcripts (DETs) in SR leaves (GN leaves as control) were detected using DEseq2 Algorithms [45] and selected based on fold change ≥ 2.0 or ≤0.5 together with adjusted *p* value (Padj) ≤ 0.05.

For certain DETs of interest, qPCR was performed to validate their transcript levels with a 7900HT Fast Real-Time PCR System (Applied Biosystems, Foster City, CA, USA) as described previously [46]. The qRT-PCR primers were designed using online Primer3 software [47] and listed in Supplementary Table S6. The qRT-PCR data were analyzed using SDS 2.2.1 software (Applied Biosystems). The relative transcript levels were normalized to a putative *UBQ* (Unigene13404_All) of *Acer palmatum*, using the equation 2^−^^ΔCt^, where Ct is the threshold cycle for each gene [48].

### 4.6. Sequence Alignment and Phylogenetic Analysis of MYB TFs

Using Clustal W of MegAlign (DNASTAR, Madison, WI, USA) with the default parameter values, peptide sequences of all putative MYB TF transcripts of *Acer palmatum* were aligned together with eleven reference MYBs with known or putative function of promoting anthocyanin biosynthesis in *Arabidopsis thaliana*, *Vitis vinifera*, *Malus domestica*, *Citrus sinensis*, *Prunus americana* and *Populus trichocarpa*. The alignment results were used to produce a neighbor-joining phylogenetic tree using MEGA5 [49]. The Bootstrap method, with 100 replications, was used to provide confidence levels (reported as a percentage) of branch points on the phylogenetic tree.

### 4.7. Isolation and Characterization of a Putative Target ApMYB1

The putative target *ApMYB1* with the function of promoting anthocyanin biosynthesis was identified from the same clade of phylogenetic tree containing reference MYBs. Total RNA was extracted from SR leaves and cDNA was synthesized. The open reading frame (ORF) of *ApMYB1* was amplified with a pair of gene-specific primers (Supplementary Table S6). The deduced peptide sequence of ApMYB1 was aligned with eleven reference MYBs to show the conserved domains. The expression of *ApMYB1* in GN and SR leaves was measured by quantitative real-time PCR (qPCR). Furthermore, the newly expanded leaves were detached from branches of *Acer palmatum* in July and were placed on filter paper wetted respectively with various solutions [including 20 μM abscisic acid (ABA), 50 μM 1-amino-cyclopropane-1-carboxylic acid (ACC, the direct precursor of ethylene), 50 μM methyl jasmonate (MJ), 100 μM salicylic acid (SA) and 1% H_2_O_2_] in petri dishes under dim light (40 μmol/s/m^2^). The treated leaves were collected at various time points, and *ApMYB1* transcript levels were measured by qPCR.

### 4.8. Stable Overexpression of ApMYB1 in Tobacco

The *ApMYB1* ORF was cloned into *pCAMBIA1305* vector via the *NcoI* and *PmlI* restriction sites to replace *GUS*plus sequence, resulting in a *35S::ApMYB1* construct for tobacco transformation. Tobacco (*Nicotiana tabacum* L. cv. Honghua Dajinyuan) was used for leaf disk transformation [50]. Primary tobacco leaf discs after Agrobacterium infection were rested on Murashige and Skoog (MS) medium (containing 15 mg/L hygromycin). Infected leaf explants were transferred onto fresh regeneration media every two weeks until shoots were regenerated. Shoots were then excised from explants carefully and transferred into rooting medium. Rooted plants were grown in pot soil in a growth room with 16 h of light at 150 photosynthetic photon flux density [μmol/(m^2^∙s^1^)] at 24 °C and 8 h of dark at 22 °C. The obtained hygromycin-resistant T1 generation plants were used for genomic DNA and total RNA extraction to confirm *ApMYB1* insertion and its expression levels. T2 seeds were collected and selected on an MS plate containing hygromycin 12 mg/L for germination. The T2 seedlings were grown in soil for phenotypic observation and anthocyanin measurement was performed.

### 4.9. Statistical Analysis

Values were presented as means ± SD of three biological replicates. Statistical difference among multiple means was first estimated by one-way variance analysis (ANOVA) and then evaluated by Duncan’s multiple range test at 0.05 probability level by using SPSS version 25.0 (IBM, Armonk, NY, USA). Significant difference between two means was analyzed by using the Student’s *t*-test at *p* < 0.05.

## Data Availability

Raw FASTQ files for the RNA-seq libraries were deposited to the NCBI Sequence Read Archive (SRA) with BioProject accession number PRJNA778962 (https://dataview.ncbi.nlm.nih.gov/object/PRJNA778962 (accessed on 11 March 2022)). Those figures and tables supporting the main results of this article are provided as the supplementary information files.

## Supplementary Materials

The following supporting information can be downloaded at: https://www.mdpi.com/article/10.3390/plants11060759/s1, Figure S1. Expression levels of selected anthocyanin biosynthesis related transcripts measured by qPCR, Figure S2. Identification of transgenic tobacco T1 plants overexpressing *ApMYB1*, Figure S3. Promoted anthocyanin biosynthesis in transgenic tobacco T2 plants (OE-20), Table S1. Differentially expressed genes, Table S2. KEGG pathway enrichment analysis, Table S3. Differentially expressed transcripts involved in anthocyanin biosynthesis, Table S4. Differentially expressed transcripts encoding putative TFs, Table S5. Protein and DNA sequences, Table S6. Primers used in this work.
