# Supplementary material for: Leaf Coloration in Acer palmatum Is Associated with a Positive Regulator ApMYB1 with Potential for Breeding Color-Leafed Plants
Source: Plants (Basel). 2022 Mar 12;11(6):759. doi: 10.3390/plants11060759 (PMC8955597; doi:10.3390/plants11060759)
Supplement: Supplementary file 1 [file plants-11-00759-s001.zip › Sun et al-Supplementary Figures S1-S3.pdf]

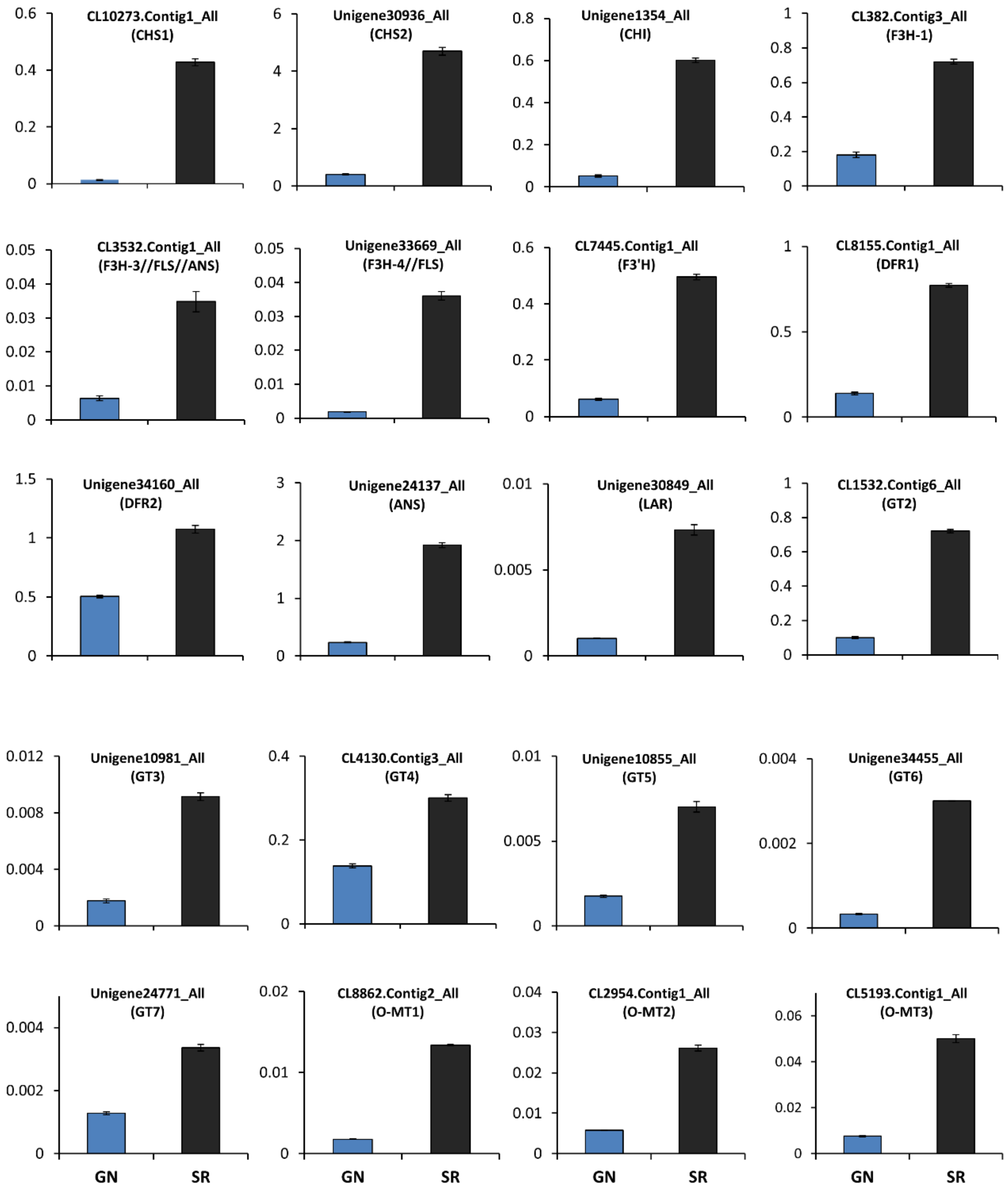

Supplemental Figure S1 Expression levels of selected anthocyanin biosynthesis related transcripts measured by qPCR. These genes were identified through RNA-Seq as up-regulated with ≥ 2 fold in SR compared to GN leaves and selected based on annotations containing names of target enzymes in anthocyanin biosynthesis pathway.

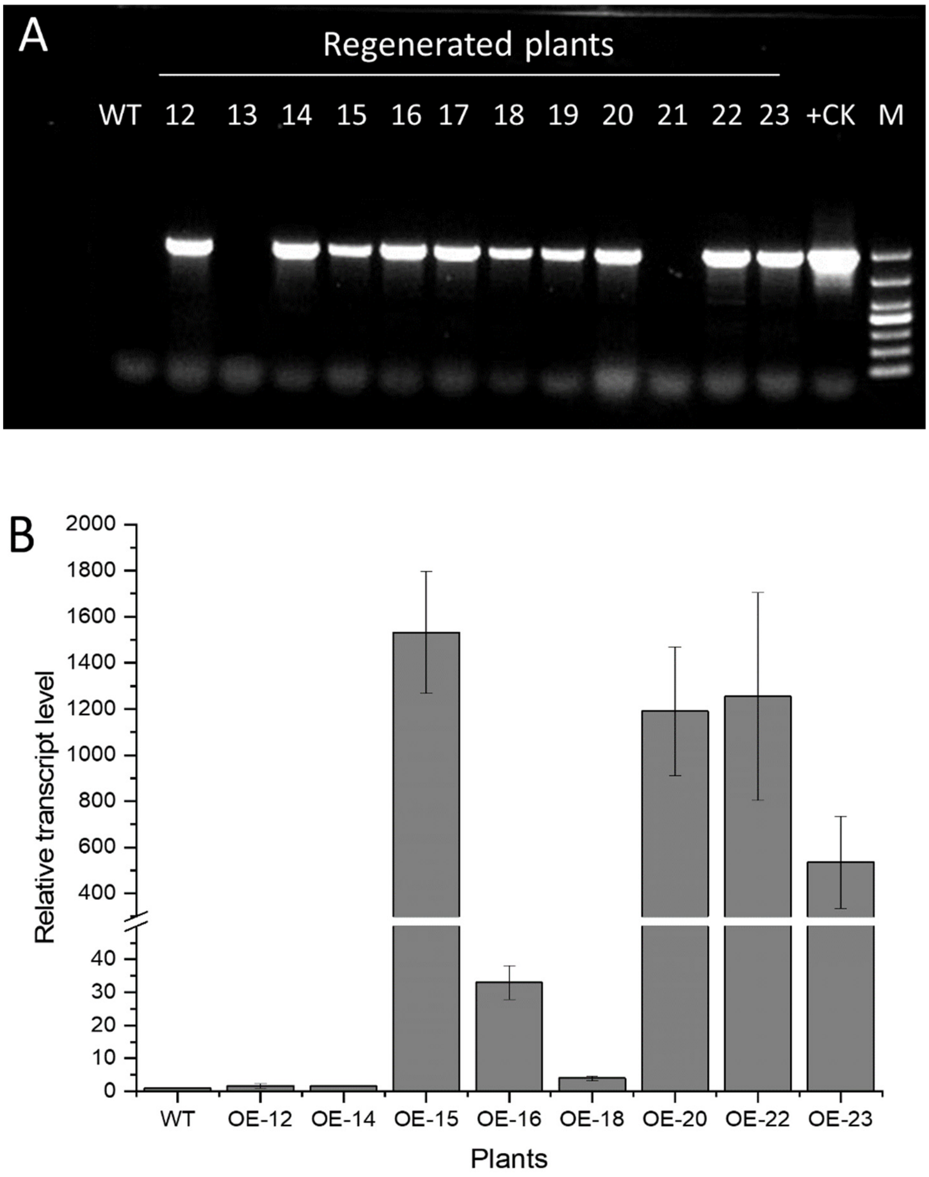

Supplemental Figure S2 Identification of transgenic tobacco T1 plants overexpressing *ApMYB1*. (A) PCR using genomic DNA of twelve regenerated tobacco T1 plants, WT (negative control) and construct plasmid (positive control) as templates. (B) Relative transcript levels of *ApMYB1* in WT and 8 transgenic positive T1 plants measured by qPCR. T1 plants of OE-20 and OE-22 were grown for T2 seed production and phenotypic observation was performed on T2 plants. The T1 plant of OE-15 was dead during growing.

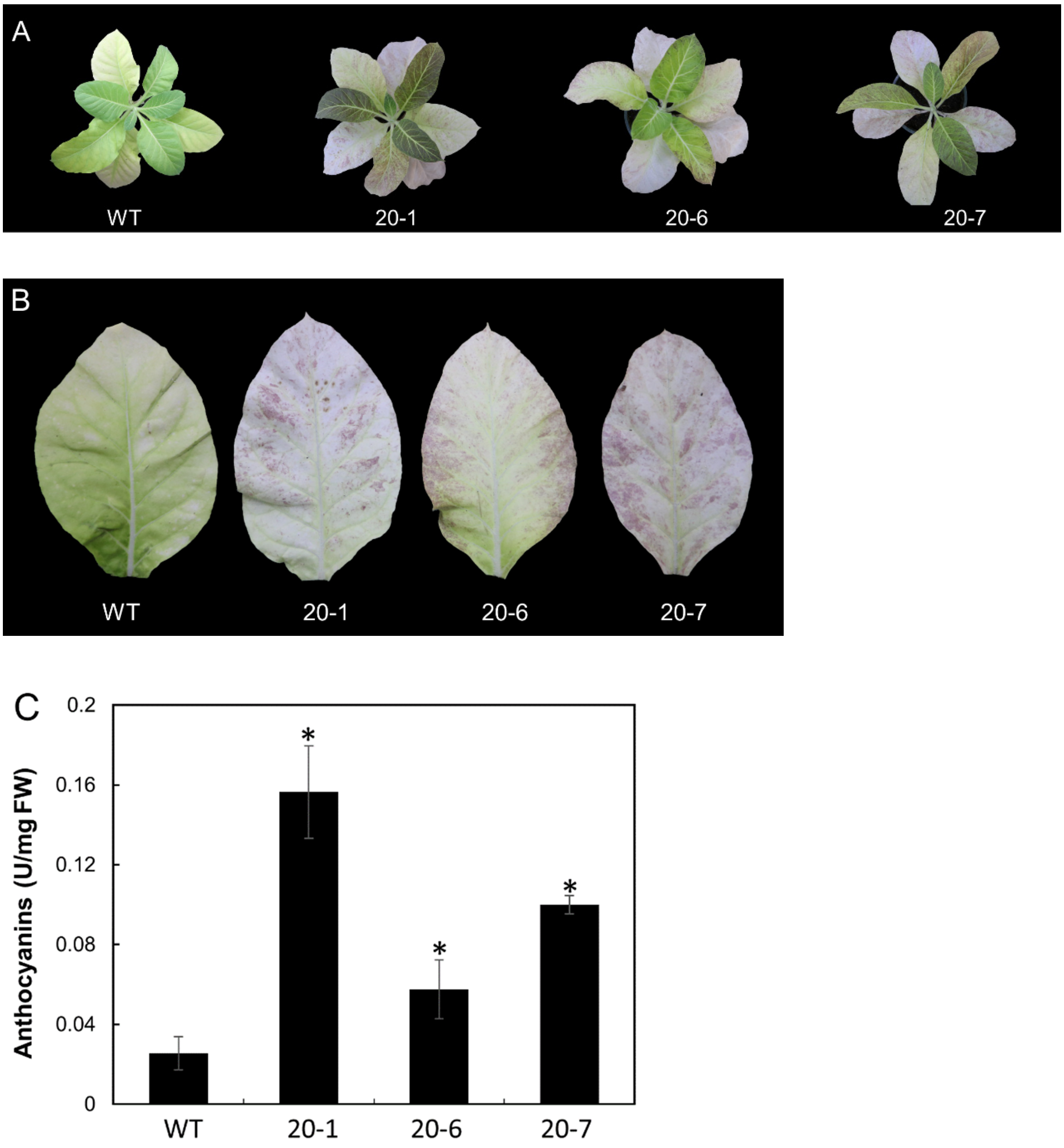

Supplemental Figure S3 Promoted anthocyanin biosynthesis in transgenic tobacco T2 plants (OE-20). (A) phenotype of WT and three T2 plants of transgenic line #20. (B) phenotype of representative leaves detached from WT and three T2 plants of transgenic line #20. (C) relative content of anthocyanins in leaves of WT and three T2 plants of transgenic line #20. Values in C are means ± SD (n=3). Asterisks indicate significant difference between various transgenic leaves and WT at $p < 0.05$, using the Student's t-test.
